# Role of Omega-3 Fatty Acids in Improving Metabolic Dysfunctions in Polycystic Ovary Syndrome

**DOI:** 10.3390/nu16172961

**Published:** 2024-09-03

**Authors:** Laila Albardan, Carine Platat, Nishan Sudheera Kalupahana

**Affiliations:** Department of Nutrition and Health, College of Medicine and Health Sciences, United Arab Emirates University, Al-Ain P.O. Box 15551, United Arab Emirates

**Keywords:** polycystic ovary syndrome, omega-3 fatty acids, metabolic syndrome

## Abstract

Polycystic ovary syndrome (PCOS) is a common endocrine disorder that impacts women of reproductive age. In addition to reproductive and psychological complications, women with PCOS are also at a higher risk of developing metabolic diseases such as obesity, type 2 diabetes and cardiovascular disease. While weight reduction can help manage these complications in overweight or obese women, many weight loss interventions have been ineffective due to weight stigma and its psychological impact on women with PCOS. Therefore, exploring alternative dietary strategies which do not focus on weight loss per se is of importance. In this regard, omega-3 polyunsaturated fatty acids of marine origin (*n*-3 PUFAs), which are known for their hypotriglyceridemic, cardioprotective and anti-inflammatory effects, have emerged as a potential therapy for prevention and reversal of metabolic complications in PCOS. Several clinical trials showed that *n*-3 PUFAs can improve components of metabolic syndrome in women with PCOS. In this review, we first summarize the available clinical evidence for different dietary patterns in improving PCOS complications. Next, we summarize the clinical evidence for *n*-3 PUFAs for alleviating metabolic complications in PCOS. Finally, we explore the mechanisms by which *n*-3 PUFAs improve the metabolic disorders in PCOS in depth.

## 1. Introduction

Polycystic ovary syndrome (PCOS) is the most common endocrine disorder among women of reproductive age with a prevalence ranging from 4 to 21% depending on the diagnostic criteria used [1]. With an onset from adolescence, this disorder presents significant reproductive and psychological challenges for women throughout reproductive life to post-menopause [2]. PCOS is typically characterized by the presence of varying degrees of high serum androgens, insulin resistance, inflammation and ovulatory dysfunction, which clinically manifest as hyperandrogenic features (hirsutism, acne and alopecia), infrequent or absent menstrual periods and polycystic ovarian morphology observed on ultrasound scan [2]. These changes can lead to heterogeneous clinical presentations ranging from subfertility to emotional distress culminating in anxiety and depression [3]. Although traditionally classified as a reproductive disorder, PCOS is now recognized as a metabolic disorder which is associated with obesity, and which increases risk for type 2 diabetes and cardiovascular disease [3].

The treatment of PCOS includes lifestyle changes and specific pharmacotherapy targeted at managing non-fertility issues and/or subfertility [2]. In overweight or obese women, weight loss induced by dietary changes reduces the level of androgen, insulin resistance and the risk for other complications [4]. While dietary strategies are effective for weight reduction in women who are overweight or obese, the widespread problem of weight stigma and its detrimental psychological effects, as well as weight regain, compromises their efficacy [2,5,6]. Further, dietary changes, even in the absence of weight loss, improve PCOS complications [2]. This is important, especially considering that some women with PCOS have a normal weight. Additionally, the great individual variability, the complexity of the PCOS syndrome, and the presence of chronic inflammation also highlight the importance of investigating other nutritional approaches that have no focus on weight loss for the management of PCOS. Women who are not overweight and adolescents would also benefit from such dietary strategies. Specific eating patterns that do not focus on weight loss and dietary bioactive compounds are two such approaches. Indeed, a myriad of dietary supplements are already used by patients with PCOS [7], albeit unsupported by clinical evidence in some instances. The most recent clinical guidelines emphasize the importance of healthcare workers providing evidence-based information to patients and incorporating complementary therapies, such as dietary supplements, in the management of PCOS, based on the wishes and beliefs of each patient [2]. Thus, it is important to explore and summarize the evidence for dietary bioactive compounds in the management of PCOS.

Several eating patterns that do not primarily focus on weight loss are effective in improving the clinical features of PCOS [2]. While several dietary bioactive compounds, such as the polyphenol resveratrol, have evidence for benefits in POCS [8], the potential teratogenicity of some of these compounds, especially in the context of women who are of reproductive age who may become pregnant, makes their applications problematic [9]. Moreover, the latest clinical guidelines highlight the importance of strategies to reduce cardiovascular disease risk in women with PCOS [2]. In this regard, omega-3 polyunsaturated fatty acids (*n*-3 PUFAs), which have a known safety record, have gained attention as a potential adjunct therapy for women with PCOS not only for reducing the clinical features of this condition but also for reducing cardiovascular disease risk. *n*-3 PUFAs are well known for their anti-inflammatory, cardioprotective and hypotriglyceridemic effects, which we have reviewed previously [10,11,12]. Consequently, they may provide a useful strategy for reducing metabolic complications associated with PCOS. Several clinical studies have demonstrated that *n*-3 PUFAs can indeed improve various components of the metabolic syndrome, including dyslipidemia, inflammation and insulin resistance in women with PCOS [13].

Despite these promising findings, there remains a gap in the synthesis of knowledge regarding the comprehensive impact of *n*-3 PUFAs on PCOS-related metabolic disorders. Furthermore, the fundamental mechanisms by which *n*-3 PUFAs achieve their advantageous effects remain incompletely understood. Filling this gap will be essential to the development of focused dietary therapies that might successfully control and possibly even reverse the metabolic problems linked to PCOS. In this review, we first outline the diagnosis, pathophysiology and management strategies of PCOS. Next, we provide a summary of the evidence demonstrating the effectiveness of different dietary approaches which do not focus on weight loss, in reducing the complications associated with PCOS. Finally, we review the clinical evidence regarding the ability of *n*-3 PUFAs to alleviate PCOS-related metabolic derangements and discuss the mechanisms by which these fatty acids improve metabolic health in women with PCOS in depth.

## 2. PCOS—Diagnosis, Pathophysiology and Management

### 2.1. Diagnosis

According to the latest diagnostic criteria proposed by the International PCOS Network [2], which is an extension of the widely used Rotterdam criteria, a diagnosis of PCOS is made if one of the three following conditions are met: (1) irregular menstrual cycles with clinical hyperandrogenism (acne, hirsutism, seborrhea or alopecia) or (2) irregular menstrual cycles with biochemical hyperandrogenism or (3) either irregular menstrual cycles or hyperandrogenism coupled with polycystic ovarian morphology on ultrasound scan [2]. As evident from this diagnosis criteria, PCOS can present in a broad spectrum of clinical features based on different pathogenic mechanisms. Thus, in order to understand how dietary patterns and bioactives can improve the reproductive and metabolic features of this condition, it is important to first understand the pathogenesis of PCOS, which is outlined below.

### 2.2. Pathophysiology of PCOS

**Figure 1:**
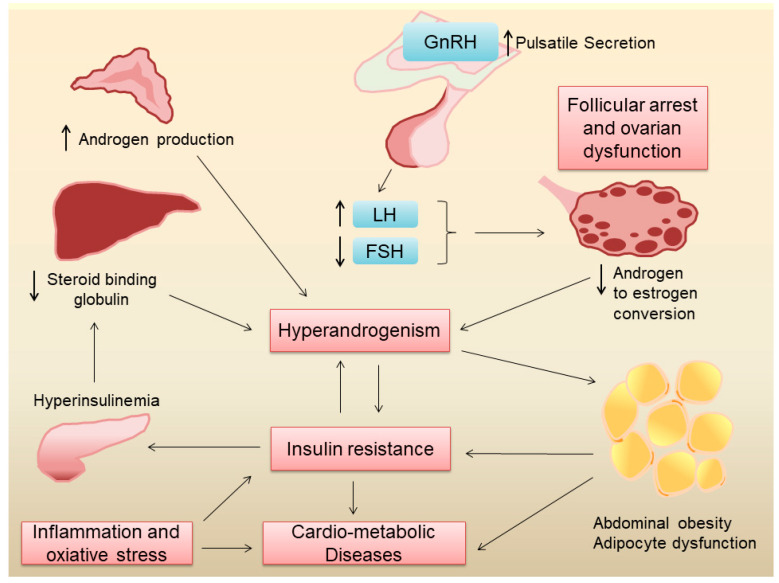
Pathogenesis of metabolic derangements in PCOS.

Women with PCOS exhibit varying degrees of hyperandrogenism, ovulatory dysfunction and insulin resistance. An intricate interplay between neuroendocrine disruptions and insulin resistance is likely responsible for the heterogeneity of PCOS presentation among women. Previous reviews have discussed these mechanisms in detail [3,14] and a brief outline is provided below and illustrated in Figure 1.

#### 2.2.1. Neuroendocrine Disruptions in POCS

The hypothalamic–pituitary–ovarian axis is the primary regulator of reproductive function in women. In eumenorrheic women, a pulsatile secretion of gonadotropin-releasing hormone (GnRH) stimulates the secretion of follicle-stimulating hormone (FSH) and luteinizing hormone (LH) from the anterior pituitary [15]. FSH stimulates the growth and maturation of ovarian follicles which is critical in normal oogenesis. LH stimulates the theca cells in the ovarian follicles to produce androgens which are subsequently converted into estrogen by the granulosa cells under the influence of FSH [15]. Since most of the androgens produced by theca cells are converted to estrogen, under normal physiological conditions, the ovaries are not a major source of androgens in women. Estrogen and progesterone produced by the ovaries, in turn, provide negative feedback to the hypothalamus and anterior pituitary, regulating the secretion of GnRH, FSH, and LH, respectively [15]. In PCOS, the pulse frequency of GnRH release by the hypothalamus is increased, which leads to a preferential release of LH over FSH from the anterior pituitary. The elevated levels of LH lead to the overproduction of androgens by ovarian theca cells, while the relatively low levels of FSH result in follicular arrest, polycystic ovarian morphology, and ovulatory dysfunction, which clinically manifests as oligomenorrhea or amenorrhea. Increased ovarian androgen production, coupled with increased production of adrenal androgens, leads to hyperandrogenism [16], which gives rise to hirsutism, acne, and alopecia in these women. While the exact cause for the changes in GnRH pulse frequency in PCOS is hitherto unknown, genetic and environmental factors seem to play an important role in this regard.

#### 2.2.2. Insulin Resistance and Hyperinsulinemia in PCOS

Several factors contribute to insulin resistance in PCOS, including genetic factors, obesity, hyperandrogenism and inflammation. Genetic studies have shown that several genes associated with insulin resistance are also linked to PCOS [17], suggesting a common origin of neuroendocrine dysregulations and insulin resistance in this condition. Around 40–80% of women with PCOS are overweight or obese [18]. Obesity, especially abdominal obesity, can contribute to insulin resistance [19] and other cardiovascular risk factors [20] in women with PCOS. Obesity also leads to adipose tissue inflammation and dysfunction, which leads to increased free fatty acids, higher pro-inflammatory cytokines, and lower anti-inflammatory adipokines, such as adiponectin, leading to systemic insulin resistance [21]. Obesity also gives rise to ectopic lipid deposition in the liver and skeletal muscle, leading to lipotoxicity, which in turn can lead to insulin resistance [22]. It is important to point out, however, that even normal-weight women with PCOS have insulin resistance [23], suggesting that adiposity-independent mechanisms may also play a role in the pathogenesis of insulin resistance in this condition. To this end, cultured fibroblasts and skeletal muscle from women with PCOS showed increased serine phosphorylation and decreased tyrosine phosphorylation of the insulin receptor, suggesting another mechanism for insulin resistance [24]. Elevated levels of androgens in PCOS can also lead to insulin resistance [25]. Chronic systemic low-grade inflammation and increased oxidative stress, which are often present in PCOS, can also lead to insulin resistance [26]. Indeed, inflammatory markers like high-sensitivity C-reactive protein (hs-CRP) and interleukins are elevated in PCOS [27], which can interfere with insulin signaling pathways. Finally, mitochondrial dysfunction, which impairs cellular energy metabolism, has been implicated in the pathophysiology of insulin resistance in PCOS [28]. It is likely that insulin resistance and chronic low-grade inflammation are major factors that increase the risk for type 2 diabetes and cardiovascular disease in women with PCOS.

While androgen excess in women with PCOS can lead to insulin resistance, the converse also holds true. In states of insulin resistance, there is compensatory hyperinsulinemia [29], which in turn can increase ovarian androgen production [30]. Hyperinsulinemia can also lead to a reduction in sex hormone-binding globulin (SHBG) production by the liver, leading to low serum SHBG levels [31]. SHBG binds to androgens and reduces their bioavailability. Thus, lower SHBG levels result in higher free androgen levels in the blood increasing their androgenic effects [32]. Taken together, these effects of insulin resistance can exacerbate hyperandrogenism in PCOS.

### 2.3. Outline of the Management of PCOS

Lifestyle interventions, comprising physical activity, healthy eating, and behavior modifications, are recommended for all women with PCOS [2]. A Cochrane database systematic review including 498 participants from 15 randomized controlled trials showed that lifestyle modification can decrease the free androgen index and body mass index in women with PCOS [33]. Lifestyle change is not only important to improve reproductive and androgenic features but also to improve metabolic health [34] and cardiovascular disease risk factors such as dyslipidemia [35,36]. While weight loss in higher-weight individuals helps to improve all components of PCOS, a healthy lifestyle, including a healthy eating pattern, can have benefits even in the absence of weight loss [2]. Dietary strategies for weight loss in women with PCOS have been studied in detail and are reviewed elsewhere [6] and are beyond the scope of the current review. A summary of dietary patterns not focusing on weight loss per se is given in the next section of this paper.

The pharmacological treatment guidelines for PCOS place a strong emphasis on shared decision-making between the patient and the practitioner, taking individual traits, values, and preferences into account [2]. Since women with PCOS can have a wide spectrum of clinical presentations, pharmacotherapy should be tailored to address individual clinical concerns [37]. A summary of pharmacotherapy for the management of non-fertility features in women with PCOS is given in Table 1.

## 3. Dietary Patterns for Preventing/Treating Metabolic Complications of PCOS

Dietary modifications significantly improve fertility, ovarian function, and clinical hyperandrogenism in women with PCOS. A meta-analysis of 20 randomized controlled trials comprising a total of 1113 women with PCOS showed that dietary modifications improved pregnancy rate and menstrual regularity with reductions in clinical features of hyperandrogenisms such as hirsutism [38]. This study further showed that dietary modification increased serum SHBG and reduced total testosterone and free androgen index. Diet also has a major role in managing metabolic complications, thereby reducing long-term cardiovascular disease risk. Since most of the beneficial effects of dietary change could be due to weight and adiposity reduction, teasing out the weight-independent effects of different eating patterns becomes problematic. However, several eating patterns, such as the Dietary Approaches to Stop Hypertension (DASH) diet, the Mediterranean diet (Med diet), and the low-glycemic index (GI) diet do not primarily focus on weight loss. In the context of prevalent weight stigma and weight bias, these dietary approaches are important tools not only for the management of reproductive and hyperandrogenic features but also to reduce the long-term cardiovascular disease risk. While the metabolic benefits of these diets in different diseases have been reviewed elsewhere [39], below, we provide a summary of the evidence for these diets in the management of PCOS.

### 3.1. Mediterranean Diet

The Med diet is an eating pattern that focuses on primarily consuming plant-based foods such as fruits, vegetables, minimally processed cereals, legumes, nuts, seeds, and olive oil. It also includes moderate amounts of dairy products, mainly fermented ones like cheese and yogurt, with low to moderate consumption of eggs, fish, and poultry, and limited red meat intake [39]. The Med diet offers significant health benefits, including lowered risks of chronic diseases such as type 2 diabetes, cardiovascular disease, and certain cancers, while also improving longevity and cognitive function [40]. In a case-control study, the PREDIMED score (indicative of the degree of adherence to this dietary pattern) showed a negative correlation with serum testosterone [41], suggesting a lowered risk of hyperandrogenism with a Med diet. Some nutrients in this dietary pattern, such as *n*-3 PUFAs, reduce inflammation and enhance insulin sensitivity, while saturated fats, which are present in high amounts in Western diets, are linked to increased testosterone levels [41]. It was observed that higher adherence to the Med diet was associated with decreased waist circumference, reduced ovarian volume, and lowered PCOS severity [42]. The cardiovascular and metabolic benefits of the Med diet are well-established. In an umbrella review of meta-analyses and observational studies showed that adherence to a Med diet reduced mortality and risk of cardiovascular disease and diabetes [43]. However, the sustainability of the Med diet faces obstacles like cultural and economic divides outside of the Mediterranean region. To overcome these challenges, accessible, reasonably priced food options and dietary standards that are culturally appropriate are needed.

### 3.2. DASH Diet

The DASH diet, well-known for its blood-pressure-lowering effects, is also beneficial for women with PCOS. Emphasizing fruits, vegetables, whole grains, lean proteins, and low-fat dairy products, the DASH diet is rich in antioxidants, magnesium, and fiber [40]. In a systematic review of 19 randomized controlled trials including 727 patients with PCOS, the DASH diet was identified as the most effective eating pattern in reducing Homeostatic Model Assessment for Insulin Resistance (HOMA-IR; a marker of insulin resistance) and serum triglycerides [44]. Another meta-analysis of 19 trials comprising 1193 participants confirmed that the DASH diet was superior to other eating patterns in improving insulin resistance in women with PCOS [45]. Other clinical studies have shown that adhering to the DASH diet leads to reductions in inflammatory markers such as hs-CRP and modest decreases in body weight and waist circumference [46,47]. By lowering insulin resistance, dysglycemia, hyperandrogenism, and excess adiposity, this diet likely enhances ovarian health and reduces metabolic dysfunction and cardiovascular disease risk in women with PCOS.

### 3.3. Low-Glycemic Index Diet

The low-GI diet, which focuses on slowly digested carbohydrates, has been shown to improve lipid profiles, insulin sensitivity, and blood glucose control, among other health outcomes [48]. This eating pattern emphasizes the intake of non-starchy vegetables, fruits, legumes, and whole grains, which elicit a low glycemic response, as well as meat, fish, poultry, eggs, and dairy products, which have a very low glycemic index. Since this eating pattern provides benefits in improving glycemic control, dyslipidemia, blood pressure, and inflammation in patients with diabetes mellitus [49], it is potentially an important dietary pattern for women with PCOS as well. Research indicates that compared to conventional diets, low-GI diets are more successful at reducing inflammation [50] and increasing insulin sensitivity [51] in women with PCOS. Low-GI diets have also been demonstrated to increase ovulation and decrease anovulation in PCOS patients [52]. In meta-analyses, low-GI diets have been linked to improved glycemic control, androgen status, and lipid profiles [53]. These diets have also been connected to better satiety, reduced overall calorie consumption, and improved weight management—all of which are critical for controlling the metabolic issues related to PCOS [53]. Nevertheless, the reliability of GI rankings, or how foods are evaluated according to their GI, has been criticized. It may not necessarily reflect the overall nutritional value of foods [48].

Dietary management guidelines emphasize that there is no ideal eating pattern for women with PCOS [2]. Any diet that complies with healthy eating recommendations is advantageous; nevertheless, to prevent restrictive and unbalanced diets, dietary modifications should be evidence-based, culturally acceptable, and customized to personal preferences [2]. When faced with obstacles in following a diet, such as psychological, physical, financial, or cultural issues, family support, and professional assistance should be taken into consideration.

In addition to healthy eating patterns, dietary bioactives such as polyphenols, flavonoids, and *n*-3 PUFAs, which have the potential to reduce inflammation and improve metabolic derangements, are important adjunct therapies in the management of women with PCOS. The most recent PCOS management guidelines emphasize that it is the responsibility of healthcare professionals to provide women with evidence-based information to make shared informed decisions, not only for pharmacotherapies but also for complementary therapies such as dietary supplements [2]. Potential benefits of dietary supplements in the management of PCOS have been previously reviewed by Iervolino et al. [54] and Alesi et al. [18]. A recent systematic review of a total of 344 studies identified 49 different dietary supplements that were used as complementary therapy for women with PCOS. Thus, it is an onerous task to provide evidence and mechanistic insights into such a wide range of supplements. In the aforementioned systematic review, the most frequently investigated supplements were inositol, vitamin D, *N*-acetylcysteine, and *n*-3 PUFAs. Since *n*-3 PUFAs have known cardiovascular benefits and a proven safety record, and since PCOS is a metabolic disorder that increases the risk of cardiovascular disease, it is important to examine the clinical as well as mechanistic evidence for the role of *n*-3 PUFAs in PCOS, which is provided in the next section of this manuscript.

## 4. Role of *n*-3 PUFAs in Preventing/Treating Metabolic Complications in PCOS

### 4.1. Sources, Biological Functions, and Side Effects of n-3 PUFAs

PUFAs are classified into *n*-3 or *n*-6 based on the location of their first double bond counting from the omega end. Both classes are considered essential fatty acids because the human body cannot synthesize sufficient amounts of these fatty acids de novo [55]. Linoleic acid (LA, 18:2) is the primary long-chain *n*-6 PUFA, which can be converted into arachidonic acid (AA, 20:4). α-Linolenic acid (ALA, 18:3) is the primary *n*-3 PUFA, which can be converted to eicosapentaenoic acid (EPA, 20:5) and docosahexaenoic acid (DHA, 22:6). LA is mainly present in vegetable oils and nuts, while the food sources of ALA include nuts and seeds such as walnuts, flaxseed and chia seeds, and vegetable oils such as flaxseed and soybean oil [56]. AA is primarily found in red meat and foods of animal origin, while marine foods, especially cold-water fatty fish such as tuna, salmon, mackerel, sardines, and herring, are the major source of EPA and DHA [56]. Detailed information on the EPA and DHA content of commonly consumed seafood is given by Rimm et al. [57].

The American Heart Association recommends the consumption of seafood one to two times per week for cardiovascular health. While adverse effects of EPA and DHA at current recommended levels are extremely rare, bleeding episodes, dysglycemia, dyslipidemia, and impaired immune function have been associated with a very high intake of EPA and DHA [58]. While no tolerable upper intake level (UL) has been set for these fatty acids, up to 5 g/day of combined EPA and DHA is considered safe by the European Food Safety Authority [58].

AA, EPA, and DHA are important components of cell membranes and are precursors of eicosanoids such as prostaglandins and leukotrienes. Further, these *n*-3 PUFAs and AA compete for the sn-2 position of membrane phospholipids, and the dietary ratio of *n*-6:*n*-3 PUFA intake, particularly the AA:EPA/DHA intake likely determines their composition in the cell membranes [59]. AA-derived eicosanoids are considered pro-inflammatory compared to EPA/DHA-derived ones. It is important to recognize that not all *n*-3 PUFAs have the same biological effects [60]. For example, the anti-inflammatory, hypotriglyceridemic, and cardioprotective effects of *n*-3 PUFA have been mainly demonstrated for EPA and DHA [61]. Similarly, the pro-inflammatory actions of *n*-6 PUFAs mainly apply to AA. Further, while LA can be converted to AA and ALA can be converted to EPA and then DHA, respectively, this conversion is inefficient in humans [59] and is unlikely to lead to high AA and EPA/DHA levels in the body. Thus, the dietary intake of individual *n*-3 or *n*-6 PUFA likely determines their biological effects in humans. Therefore, it is important to consider the type of *n*-3 PUFA, as well as the dose used, when interpreting findings from clinical studies using *n*-3 PUFA supplementation.

### 4.2. Efficacy of n-3 PUFAs in Improving Metabolic Markers in PCOS

A case-control study of 325 pairs of women with PCOS and healthy controls from China showed that women in the highest tertile of serum phospholipid *n*-3 PUFAs were 40% less likely to have PCOS compared to those in the lowest tertile [62]. Further, the same study found an inverse correlation between both dietary and serum long-chain *n*-3 PUFAs with serum insulin, total testosterone, and hs-CRP, and a positive correlation with serum FSH and SHBG [62], suggesting a role for *n*-3 PUFA in reducing the risk of PCOS. Another cross-sectional study, which included 185 women with PCOS from China, found that both dietary *n*-3 PUFA intake as well as serum *n*-3 PUFA, especially EPA and DHA levels, were negatively correlated with HOMA-IR index and body fat levels [63], suggesting that *n*-3 PUFA may help in improving insulin resistance among these women.

Several clinical trials have investigated the efficacy of *n*-3 PUFA in improving metabolic markers in women with PCOS, some of which are summarized in Table 2. In a meta-analysis of nine randomized controlled studies including 591 women with PCOS, *n*-3 PUFA decreased HOMA-IR, serum total cholesterol, and triglycerides [13]. The magnitude of reduction in triglycerides was especially noteworthy (mean reduction of 29.21 mg/dl with a 95% confidence interval of −48.08, −10.34), suggesting that *n*-3 PUFA may be useful for PCOS patients with high serum triglyceride levels. Another recent meta-analysis of 11 randomized controlled trials with 816 women with PCOS showed similar beneficial effects of *n*-3 PUFA, especially in trials with a duration longer than 8 weeks, in improving HOMA-IR, serum triglycerides, cholesterol, and adiponectin [64].

While most clinical studies (Table 2) and meta-analyses [13,64,65,66] summarizing data from those studies consistently show that *n*-3 PUFAs are beneficial in improving insulin resistance and dyslipidemia associated with PCOS, these studies also highlight that there is only a modest or no effect of *n*-3 PUFA in weight loss in these women. In summary, *n*-3 PUFAs have the potential to ameliorate metabolic issues linked to polycystic ovary syndrome (PCOS), such as insulin sensitivity, lipid metabolism, and hormonal balance.

**Table 2:** Summary of clinical studies on the effect of omega-3 fatty acids on metabolic markers in PCOS.

Study	Sample Size	Duration	Study Design	Intervention	Key Findings
[67]	25	8 weeks	Randomized cross-over study	EPA + DHA (3.3 g/day) vs. olive oil	Reduced hepatic fat content, serum triglycerides, and systolic and diastolic blood pressure compared to control
[68]	22	6 weeks	Randomized cross-over study	EPA + DHA (2.4 g/day) vs. olive oil	Reduced plasma bioavailable testosterone compared to control
[69]	64	8 weeks	Randomized controlled study	EPA + DHA (1.2 g/day) vs. placebo	Increased plasma adiponectin, decreased insulin, glucose, HOMA-IR, total and LDL-cholesterol compared to control; no change in hs-CRP
[70]	61	8 weeks	Randomized controlled study	EPA + DHA (1.2 g/day) vs. Placebo	Decreased glucose, insulin, and HOMA-IR; no change in anthropometry
[71]	68	12 weeks	Randomized controlled study	ALA (400 mg) + Vitamin E (400 IU) vs. Placebo	Reduced *LPA* expression in blood mononuclear cells, reduced serum total and LDL cholesterol, triglycerides, and VLDL cholesterol; increased serum total antioxidant capacity
[72]	40	12 weeks	Randomized controlled study	Fish oil (1 g/day) vs. placebo	Up-regulation of *PPARG* and down-regulation of *IL1A* and *CXCL8* (IL-8) in peripheral blood mononuclear cells
[73]	60	12 weeks	Randomized controlled study	Fish Oil (2 g/day) + Vitamin D 50,000 IU bi-weekly or Placebo	Decrease in hs-CRP and malondialdehyde, increase in total antioxidant capacity
[74]	60	12 weeks	Randomized controlled study	Flaxseed oil (2 g/day) or placebo	Decrease in insulin, HOMA-IR, serum triglycerides, VLDL cholesterol and hs-CRP
[75]	60	12 weeks	Randomized controlled study	Flaxseed oil (1 g/day containing 400 mg ALA) + Vitamin E (400 IU) or placebo	Decreases in carotid intima-media thickness and hs-CRP levels
[76]	51	6 weeks	Randomized controlled study	Fish oil (EPA + DHA 3.5 g/day) or flaxseed oil (ALA 3.5 g/day) or placebo of soybean oil	Reduced triglycerides by both fish oil and flax seed oil
[77]	84	8 weeks	Randomized controlled study	Fish oil (EPA + DHA 900 mg) or placebo	Reduced LH and increased adiponectin by fish oil
[78]	88	6 months	Randomized controlled study	Fish oil (EPA + DHA 600 mg/day) vs. placebo	Decreased waist circumference, serum LDL-C, triglycerides, total cholesterol, and menstrual cycle interval; increased HDL-C by fish oil
[79]	45	6 months	Randomized controlled study	*n*-3 PUFA (1.5 g/day) vs. placebo	Decreased BMI, serum insulin, HOMA-IR, hirsutism score, serum testosterone, and LH; increased SHBG by fish oil

### 4.3. Potential Mechanisms by Which n-3 PUFAs Improve Metabolic Markers in PCOS

#### 4.3.1. Reducing Serum Triglycerides

Numerous randomized controlled trials (Table 2) and meta-analyses of those trials [13,65] have shown that *n*-3 PUFAs (either ALA or EPA/DHA) are effective in reducing serum triglycerides in women with PCOS. The triglyceride-lowering effects of *n*-3 PUFAs, particularly EPA and DHA, are well-established [80]. Further, the REDUCE-IT trial showed that high-dose EPA ethyl ester (icosapent ethyl—4 g/day) reduced major cardiovascular events in patients with existing cardiovascular disease or diabetes and elevated plasma triglycerides [81], confirming that the triglyceride-lowering effects of these fatty acids also extend into cardio-protection in the longer term. Mechanistically, *n*-3 PUFAs reduce plasma triglycerides by increasing fatty acid oxidation, thereby inhibiting hepatic lipogenesis and VLDL cholesterol production [80]. Although these mechanisms have not been studied in women with PCOS, it is likely that the same operation is present in this metabolic disorder as well.

#### 4.3.2. Reducing Androgen Production

**Figure 2:**
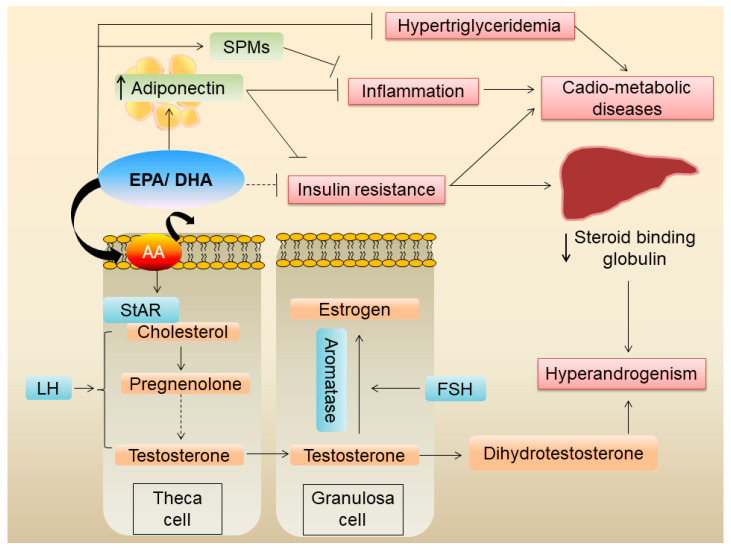
Mechanisms by which EPA/DHA improve metabolic derangements in PCOS.

A cross-sectional study of 104 white pre-menopausal women with PCOS from Australia showed that a higher plasma concentration of *n*-6 PUFA and a higher *n*-6:*n*-3 ratio was linked to increased circulating androgens [68]. A subsequent randomized cross-over study of 22 women conducted by the same investigators showed that daily 2.4 g of EPA + DHA supplementation for six weeks significantly reduced plasma bioavailable testosterone levels [68]. Mechanistically, the reduced testosterone in the *n*-3 PUFA group is likely to be due to a reduced *n*-6:*n*-3 ratio leading to reduced availability of AA in cell membranes (Figure 2), since *n*-6 PUFA treatment was shown to increase androstenedione secretion from bovine theca cells in vitro [68]. Previous studies have shown that AA is important for LH-stimulated steroidogenesis in vitro [82]. This AA effect is at least in part via the regulation of steroidogenic acute regulatory protein (StAR) expression [83]. From the clinical studies, a reduction in serum testosterone was mainly seen with supplementation of fish oil (EPA + DHA) rather than flax seed oil (ALA). As mentioned before, EPA and DHA directly compete with AA for incorporation to cell membranes, while ALA indirectly reduces AA via competing with delta 6 desaturase, the enzyme which converts LA to AA [84]. Thus, fish oil (EPA/DHA) appears to be more effective in reducing serum testosterone in women with PCOS rather than ALA-containing supplements such as flax seed oil.

It is important to point out, however, that the dietary or plasma *n*-6:*n*-3 ratio might not be the best indicator of the AA and EPA/DHA levels in the cell membranes. For example, a change (increase or decrease) in dietary LA does not correlate with changes in the AA level in the serum phospholipid pool [85]. Therefore, increasing dietary intake of EPA/DHA and/or decreasing dietary AA is likely important for a change in the lipid composition of the cell membranes leading to a reduced bioavailability of AA. Further, studies using the omega-3 index (a measure of EPA + DHA in red blood cells) or specific AA/EPA + DHA levels in serum phospholipids and their relation to the preventive/therapeutic aspects of PCOS are warranted.

A meta-analysis of ten randomized controlled trials including 610 patients found that *n*-3 PUFA decreases serum total testosterone and increases SHBG in women with PCOS [86]. Although the exact mechanism of increase in SHGB following *n*-3 PUFA is not clear, it is possible that it could be an indirect effect due to improved insulin sensitivity.

#### 4.3.3. Anti-Inflammatory Effects of *n*-3 PUFA

The same meta-analysis mentioned above in Section 4.3.2 reported that *n*-3 PUFA reduces serum hs-CRP and malondialdehyde while improving serum total antioxidant capacity [86]. Another meta-analysis of 10 randomized controlled trials, including 778 women with PCOS, also showed a reduction in serum hs-CRP following *n*-3 PUFA supplementation [66]. In a case-control study of 31 Iranian women with PCOS and 29 matched-controls, plasma-resolving D1 level showed a strong negative correlation with the HOMA–IR index in both women with PCOS as well as controls [87] suggesting that this DHA-derived specialized pro-resolving mediator (SPM) may be important in improving insulin resistance in women with PCOS. Resolvins, protectins, and maresins are EPA/DHA-derived SPMs, which enhance the resolution phase of inflammation [88]. Detailed effects of SPMs on PCOS have been reviewed by Regidor et al. previously [89].

PCOS is associated with chronic low-grade inflammation and increased oxidative stress. A meta-analysis of 10 randomized clinical trials showed that *n*-3 PUFA supplementation decreased serum hs-CRP and increased serum adiponectin levels [90], confirming the benefit of *n*-3 PUFAs in reducing systemic inflammation in women with PCOS. However, this study did not find a change in malondialdehyde levels or total antioxidant capacity, suggesting that *n*-3 PUFA may not exert antioxidative effects. An umbrella review of 28 meta-analyses of randomized controlled trials on nutrient interventions in PCOS showed that fish oil supplements increased (with a high level of certainty) serum adiponectin levels [65]. The effects of EPA/DHA on serum adiponectin are well-established. A meta-analysis of 14 randomized controlled trials showed that fish oil significantly increased serum adiponectin levels [91]. Since adiponectin is an anti-inflammatory adipokine and a known insulin sensitizer, the beneficial effects of *n*-3 PUFA, mainly EPA/DHA, on inflammation and insulin resistance is likely at least in part mediated via increased serum adiponectin.

*n*-3 PUFAs of marine origin have known anti-inflammatory effects on adipose tissue [12]. Since obesity frequently co-exists in women with PCOS, and since hyperandrogenism itself can lead to abdominal obesity and adipose tissue dysfunction (Figure 1), it is plausible that these fatty acids reduce adipose tissue inflammation. Further, EPA/DHA are potent activators of the G protein-coupled receptor 120 (GPR120), which has potent anti-inflammatory effects, particularly against macrophage-induced tissue inflammation [92]. Whether *n*-3 PUFAs exert similar anti-inflammatory effects on adipose tissue in women with PCOS has not been shown and warrants further investigation.

#### 4.3.4. *n*-3 PUFAs and Gut Dysbiosis

In a DHEA-induced PCOS mouse model, eight weeks of *n*-3 PUFA supplementation was able to reverse the DHEA-induced increases in serum LH levels as well as insulin resistance and ovarian dysfunction [93]. The latter effect was likely due to the anti-inflammatory effect of *n*-3 PUFA in the ovaries. Since antibiotic-treated mice also had similar improvements by *n*-3 PUFA, it is unlikely that the *n*-3 PUFA-mediated improvements in metabolic markers are gut microbiota-dependent. However, since the fecal transfer from *n*-3 PUFA-treated mice to PCOS mice resulted in improvements in adipocyte morphology and function [93], a gut microbiota-adipocyte crosstalk modulated by *n*-3 PUFA may be possible. In another study using a letrozole-induced PCOS rat model, fish oil supplementation ameliorated the defects in ovarian morphology and estrous cycle, which was associated with reduced ovarian and systemic inflammatory markers, serum LPS levels, and a favorable change in gut microbiota [94]. This study also suggests a possible role of gut microbiota in the *n*-3 PUFA-mediated improvements of PCOS. Clinical studies and further mechanistic studies on the *n*-3 PUFA–gut microbiota interaction in PCOS are warranted.

## 5. Conclusions

In addition to being a reproductive disorder, PCOS is now recognized as a metabolic disease that increases the risk of type 2 diabetes and cardiovascular disease. Accordingly, the latest clinical guidelines emphasize the importance of prioritizing strategies to lower cardiovascular risk in women with PCOS [2]. Moreover, in the context of the widespread prevalence of weight stigma and weight bias, there is a reluctance of healthcare workers as well as patients to discuss diet. Therefore, dietary strategies which do not focus primarily on weight loss, and which also reduce cardio-metabolic disease risk are of importance. In this regard, the DASH diet, Med diet, and low-GI diet are evidence-based and have been shown to improve features of PCOS. Moreover, since these eating patterns have been well-established for their beneficial effects on reducing cardiovascular disease risk factors, these diets are important in lifestyle change in women with PCOS. Prospective cohort studies or longer-duration clinical trials are necessary to specifically identify if these diets are effective in reducing cardiovascular events in women with PCOS.

*n*-3 PUFAs, particularly those of marine origin (EPA and DHA), reduce serum triglycerides and improve insulin resistance and inflammation in women with PCOS. Considering the relative safety of these supplements, they could be used as an important adjunct, especially when personalizing the management based on existing PCOS phenotypes and cardio-metabolic risk. Future studies should pay specific attention to the doses and types of *n*-3 PUFA used, the different ethnic groups studied, different phenotypes of PCOS, and baseline metabolic profiles of the participants. While there is clinical evidence, only a few mechanistic studies are available to dissect the mechanisms by which *n*-3 PUFAs improve components of PCOS. Especially, the mechanisms by which *n*-3 PUFAs reduce androgen production and increase SHBG in POCS warrant further research. Such studies may help to identify novel therapeutic targets for this metabolic disease.

## Figures and Tables

**Table 1:** Summary of pharmacological management of non-fertility features of PCOS (adapted from [2].)

Treatment	Description
Combined oral contraceptive pills	For managing hirsutism and irregular menstrual cycles in women and adolescents with PCOS.
Metformin	Suggested to improve metabolic outcomes in persons with PCOS and a BMI > 25 kg/m^2^. May be explored for cycle control in teenagers and individuals with a BMI < 25 kg/m^2^ despite weak data. Should be part of lifestyle changes with low-dose administration to reduce adverse effects.
Combination therapy	Combining COCP with metformin offers little additional benefit over either medication alone, except in high metabolic risk groups.
Inositol	For improving metabolic profile.
Anti-obesity drugs	For managing greater weight in PCOS patients, lifestyle therapies and medications such as orlistat, semaglutide, liraglutide, and GLP-1 receptor agonists may be combined.
Anti-androgens	May be used to treat hirsutism in cases where COCP and cosmetic therapy are ineffective, when used with effective contraception.
